# Supplementary material for: Nanoscale imaging of CD47 informs how plasma membrane modifications shape apoptotic cell recognition
Source: Commun Biol. 2023 Feb 22;6:207. doi: 10.1038/s42003-023-04558-y (PMC9947010; doi:10.1038/s42003-023-04558-y)
Supplement: Supplementary file 2 — Supplemental Information [file 42003_2023_4558_MOESM2_ESM.pdf]

Supplemental figures S1-S10

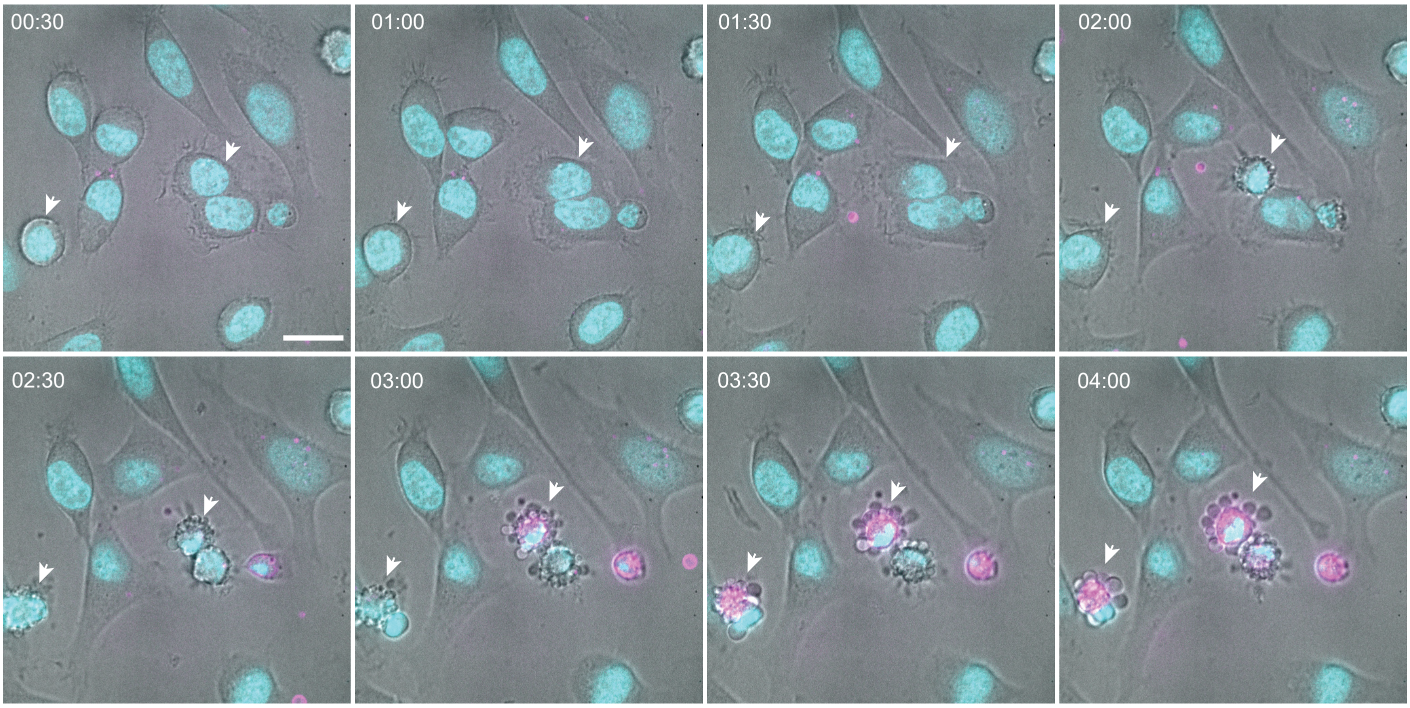

Figure S1. Characterization of UVB-induced apoptosis of HeLa cells.
HeLa cells were irradiated with UVB and then stained with Hoescht 33342 (cyan) to detect the nucleus and imaged in the presence of Alexa fluor 647-labeled Annexin V (magenta) to detect phosphatidylserine exposure. Cells were visualized during 4 hours after irradiation with a spinning disk confocal microscope to create a time-lapse image series of apoptotic HeLa cells. The shown images were selected every 30 minutes as indicated. Arrows point to cells undergoing typical membrane shrinkage and blebbing, which were used as criteria for the selection of apoptotic cells in STORM and SPT experiments. The first step is cell rounding resulting from cell retraction (2:00). After that, apoptotic membrane blebbing begins, which involves the formation of circular bulges at the plasma membrane (2:30). Then at a later stage, drastic changes in cell shape occur that are called dynamic blebbing (3:00–4:00). Scale bar 20 µm.

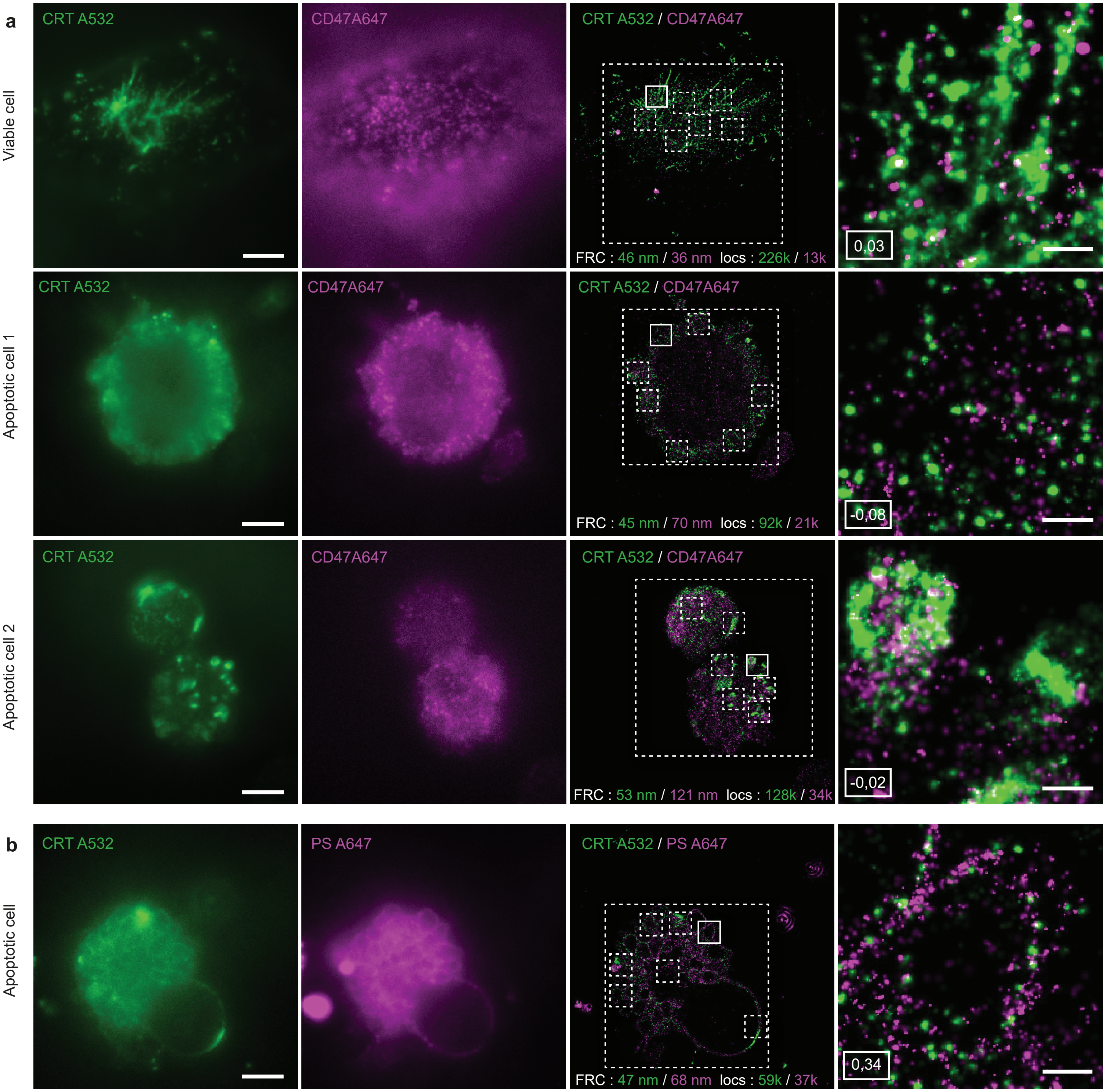

Figure S2. STORM images details and their corresponding widefield views.
STORM images shown in Figure 1 with their corresponding widefield (WF) views imaged at low laser power. (a) Viable and apoptotic cells labeled for CRT and CD47 detection. (b) Apoptotic cell labeled for PS and CRT detection. Regions selected to perform Spearman's rank correlation analysis (as shown in Figure 1e and Figure S5) are represented on the two-color images. The solid squares correspond to the zoomed views on the right. The FRC (Fourier Ring Correlation) used to evaluate the resolution for both labels and the number of localizations (k = 1000) are indicated at the bottom of the two-color STORM images. The corresponding Spearman rank correlation value is presented at the bottom of the zoomed views. Scale bars: 5μm on WF and STORM images; 500 nm on zoomed views.

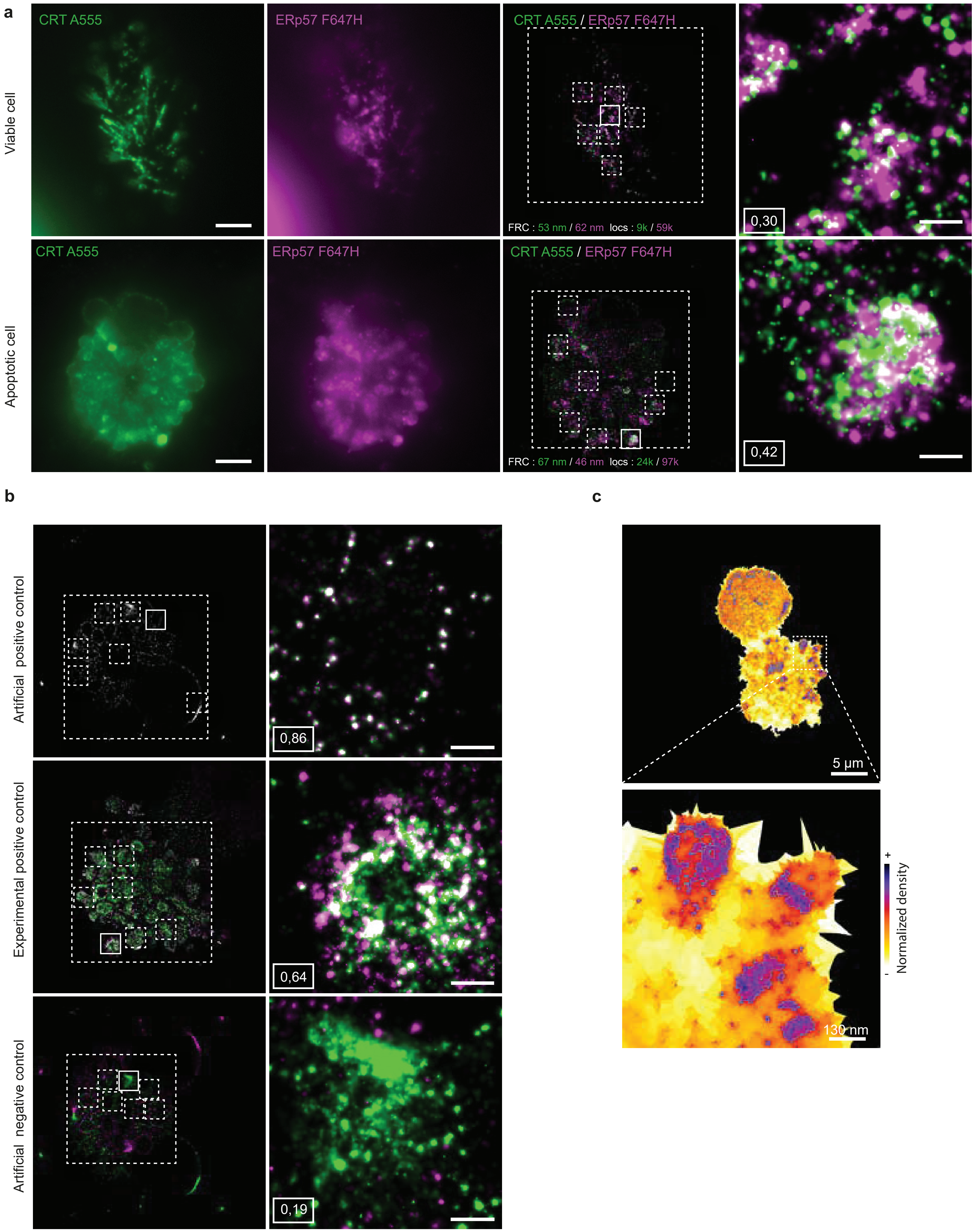
a
Viable cell
Apoptotic cell
CRT A555
ERp57 F647H
CRT A555 / ERp57 F647H
FRC : 53 nm / 62 nm locs : 9k / 59k
0,30
FRC : 67 nm / 46 nm locs : 24k / 97k
0,42
b
Artificial positive control
0,86
Experimental positive control
0,64
Artificial negative control
0,19
c
5 µm
130 nm
Normalized density
+
-

Figure S3. Images used for calibration of the Spearman rank correlation analysis, cluster sizes measurements and analysis of co-localization on selected ROIs.

(a) ERp57/CRT colocalization. ERp57 (magenta) and calreticulin (green) were detected with anti-ERp57 (ab10287) and anti-calreticulin (PA1-902A) antibodies and revealed by secondary antibodies labeled with FluoProbes 647H and Alexa Fluor 555 fluorophores respectively. (b) The artificially created positive control was obtained after randomly splitting the localizations from the CRT A532-image of Figure 1d to create two channels with colocalized fluorophores. The experimental positive control was obtained by staining calreticulin with an anti-calreticulin antibody (PA1-902A) and two distinct secondary antibodies labelled with A532 and A647 fluorophores, respectively. The artificial negative control was obtained after flipping vertically the localizations from the CRT A532-image of Figure 1e to create two channels with non co-localized fluorophores. Selected regions to perform Spearman's rank correlation analysis represented in (d) are shown on the two-color images. The solid squares corresponds to the zoomed views. The Spearman's rank correlation values are indicated at the bottom of the zoomed views. Scale bars, 5µm on WF and STORM images, 500 nm on zoomed views. (c) Images show the density maps of the apoptotic cell of Figure 1c computed with Voronoï diagrams and normalized by the average localization density. Inset shows zoomed views of apoptotic blebs, with cyan lines representing the cluster contours.

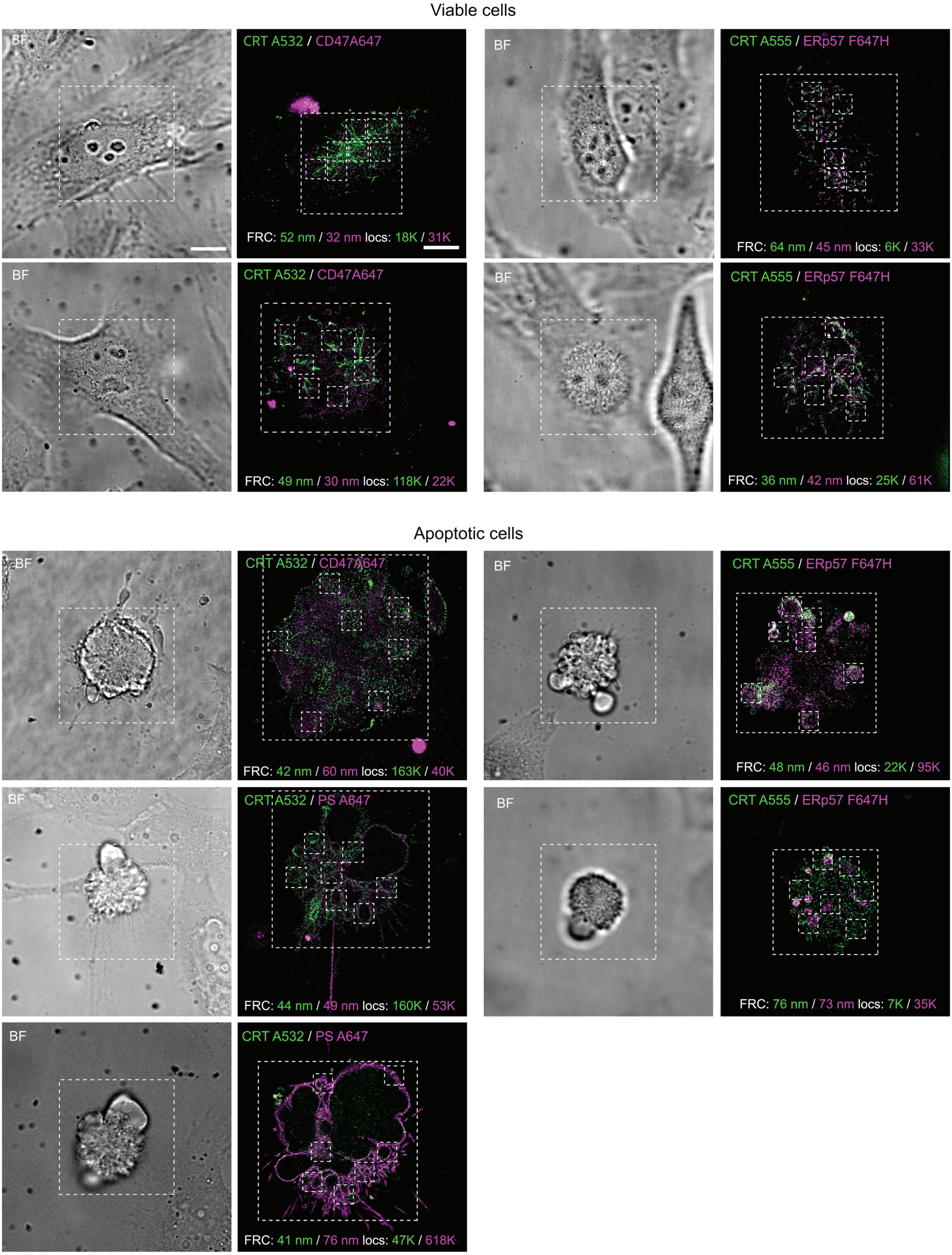

Figure S4. Additionnal 2D STORM images acquired for co-localization studies.
Bright fields (BF) and two-color images with selected ROIs used to analyse colocalization in selected areas used for figure S4b in addition to those shown on figure 1a-d and S2. The FRC (Fourier Ring Correlation) used to evaluate the resolution for both label and number of localizations (k = 1000) are indicated at the bottom of the two-color STORM images. Scale bars 10µm (BF) and 5µm (STORM).

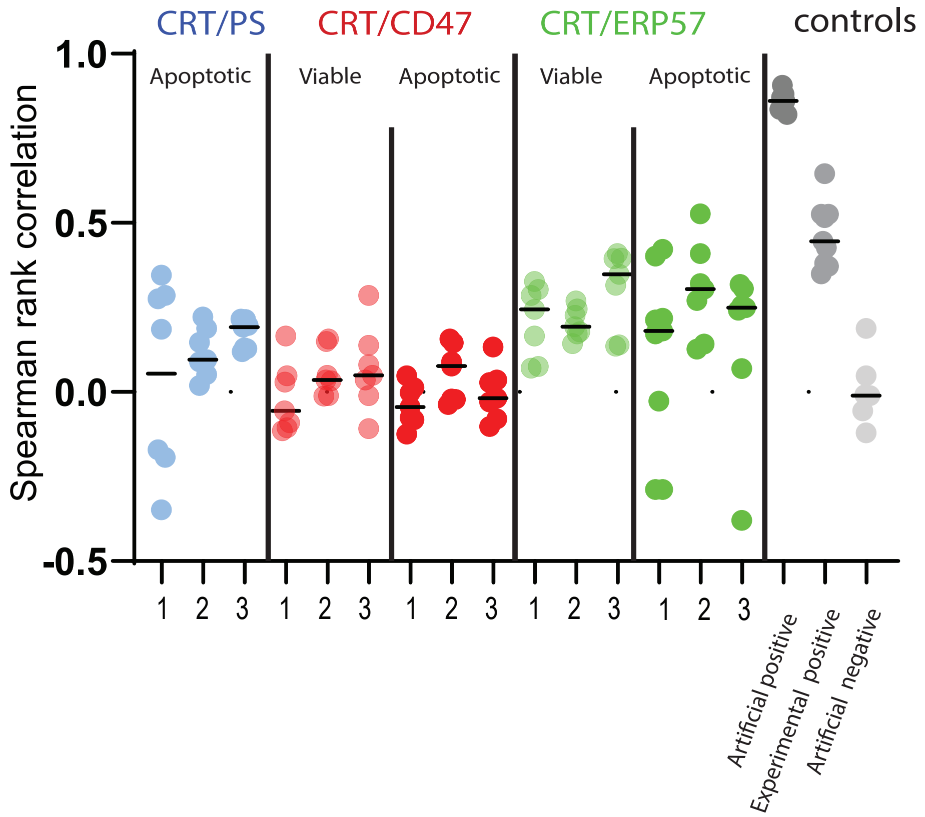

Figure S5. Analysis of Spearman's rank correlation done on selected ROIs.
Areas of 2.6 µm² (n≥7 per cell) were selected from three cells (1, 2, 3) cells among those analyzed in fig1e and shown in figures S2 and S3. Each dot corresponds to one area. Means are shown. Experimental, artificial positive and artificial negative controls were obtained as described in the methods section and represented in Figure S4a and b. Overall, this denotes that the heterogeneity observed at the whole cell level (Figure 1e) can also be found locally when analyzing different regions of a cell.

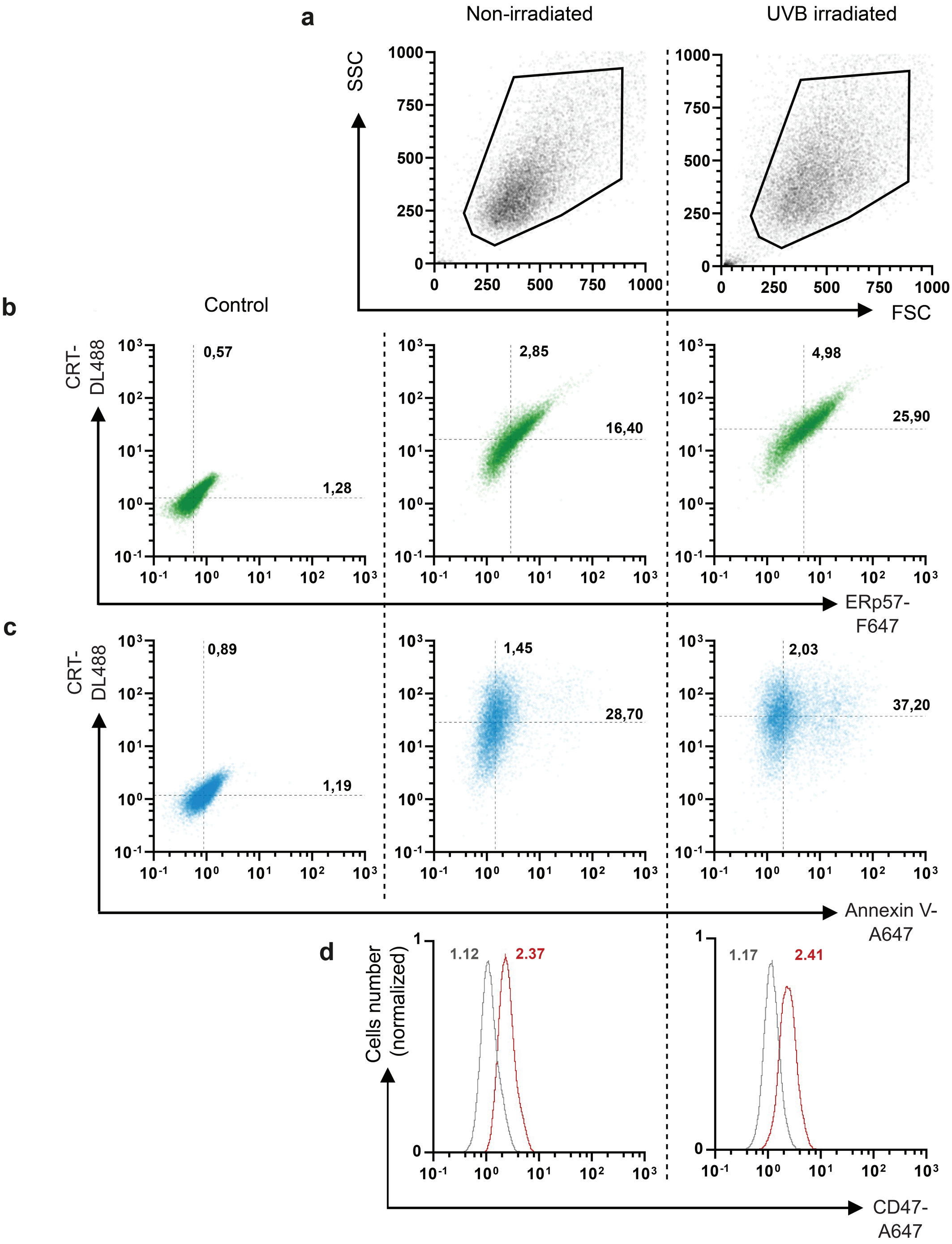

Figure S6. Flow cytometry analysis of CRT, ERP57, PS and CD47 exposure on viable and apoptotic cells
CRT and ERp57 are significantly exposed at the surface of viable HeLa cells and increased after UVB irradiation. CRT exposure precedes PS exposure and CD47 does not significantly evolve on UVB irradiated cells. HeLa cells were irradiated or not with UVB and fixed 4 hours after irradiation. CRT, ERp57, PS and CD47 were detected with anti-calreticulin antibodies (PA1-902A), anti-ERp57 antibodies (ab10287), annexin V labeled with A647 and anti-CD47 antibodies (B6H12 labelled with A647). CRT and ERp57 primary antibodies were revealed by secondary antibodies labelled with DL488 and F647 fluorophores respectively. (a) SSC/FSC dot plots for non-treated cells (Non-irradiated) or cells harvested 4 hours after UVB irradiation. (b) The corresponding dot plots for CRT/ERP57 detection and (c) for CRT detection/Annexin V binding with their negative controls on the left. (d) Histogram representation of CD47 detection (red) with its corresponding control (gray) in each condition. Median fluorescence intensity values for each channel are annotated. Experiments were performed at least two times.

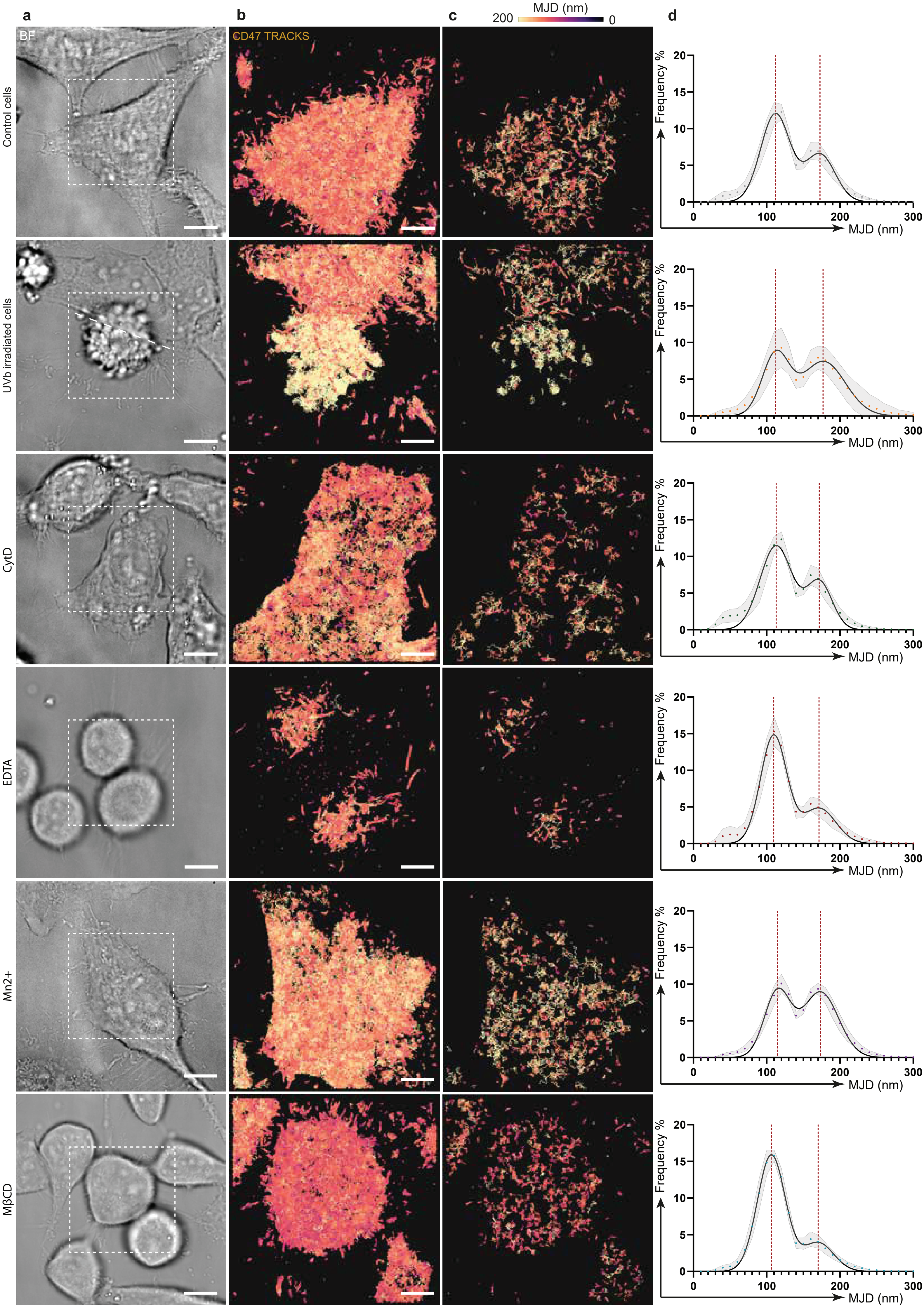

a
b
c
d
BF
CD47 TRACKS
MJD (nm)
200
0
Control cells
UVb irradiated cells
CytD
EDTA
Mn2+
MβCD
Frequency %
MJD (nm)
0
100
200
300
0
5
10
15
20

Figure S7. Detailed measurements of the MJD distribution analyzed after treatment
(a-d) CD47 was detected with an anti-CD47 (B6H12) antibody labeled with A647 and imaged by TIRF microscopy during 120 s at 33 Hz. CD47 was visualized on non-treated cells (control), cells irradiated by UVB, or cells treated with Cytochalasin D (CytD), EDTA, Mn2+ and MβCD. (a) Brightfield images were used to monitor the status of the cell. Mean Jump Distances (MJDs) were calculated and each CD47 trajectory was colored according to its MJD. Two densities were represented corresponding to 4000 (b) and 250 (c) frames interval. (d) MJD mean distributions used in (Figure 3c) represented as histograms. Dotted red lines correspond to the MJD mean value of the low and high mobility populations respectively: 111.9 nm and 172.7 nm for control cells, 111.7 nm and 176.9 nm for control UVB irradiated cells, 112.9 nm and 171.7 nm for CytD treated cells, 109.6 nm and 171.4 nm for EDTA treated cells, 114.7 nm and 173.4 nm for Mn2+ treated cells and 106.4 nm and 170.2 nm for MβCD treated cells. Dots represent the mean and the gray surface corresponds to the standard deviation. Control cell and UVB irradiated cells (a,b) are also represented in Figure 3. Scale bar, 10 µm on BF images, 5 µm on CD47 tracks reconstructed images.
.

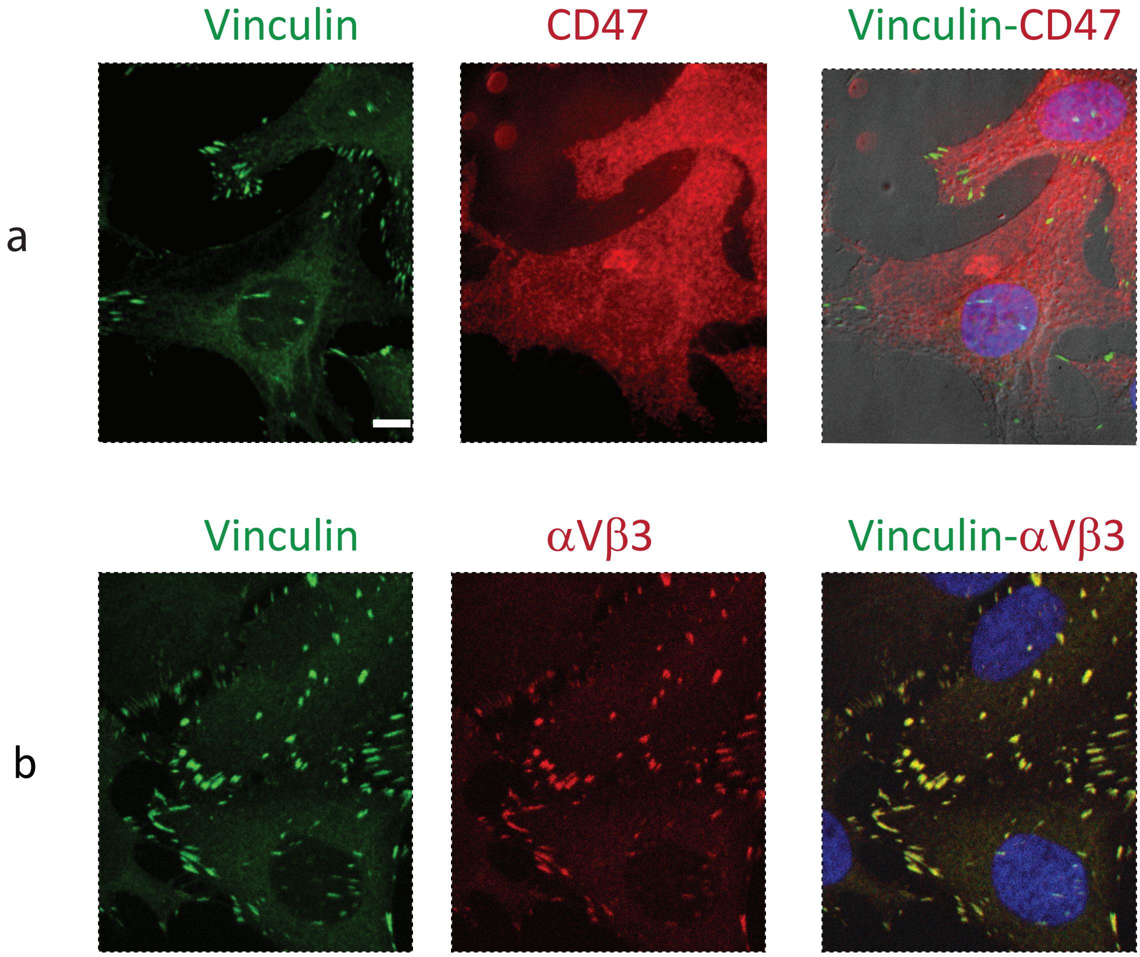

Figure S8. Homogenous distribution of CD47 in contrast to αVβ3 integrin which colocalizes with vinculin in focal adhesion sites.

CD47, vinculin and αVβ3 integrin were immunolabeled as follows: cells were fixed with 3% paraformaldehyde and 0.1% glutaraldehyde,permeabilized by saponin 0.05% and then incubated with dye -labeled antibodies. a) anti-vinculin (7F9-A488) and antiCD47 (B6H12-A647) b) anti-vinculin (7F9-A488) and anti-αVβ3 (23C6-PE). All samples were imaged in Fluoromount G with DAPI under a laser spinning-disk microscope as described in the materials and methods section. Scale bar 10 μm.

.

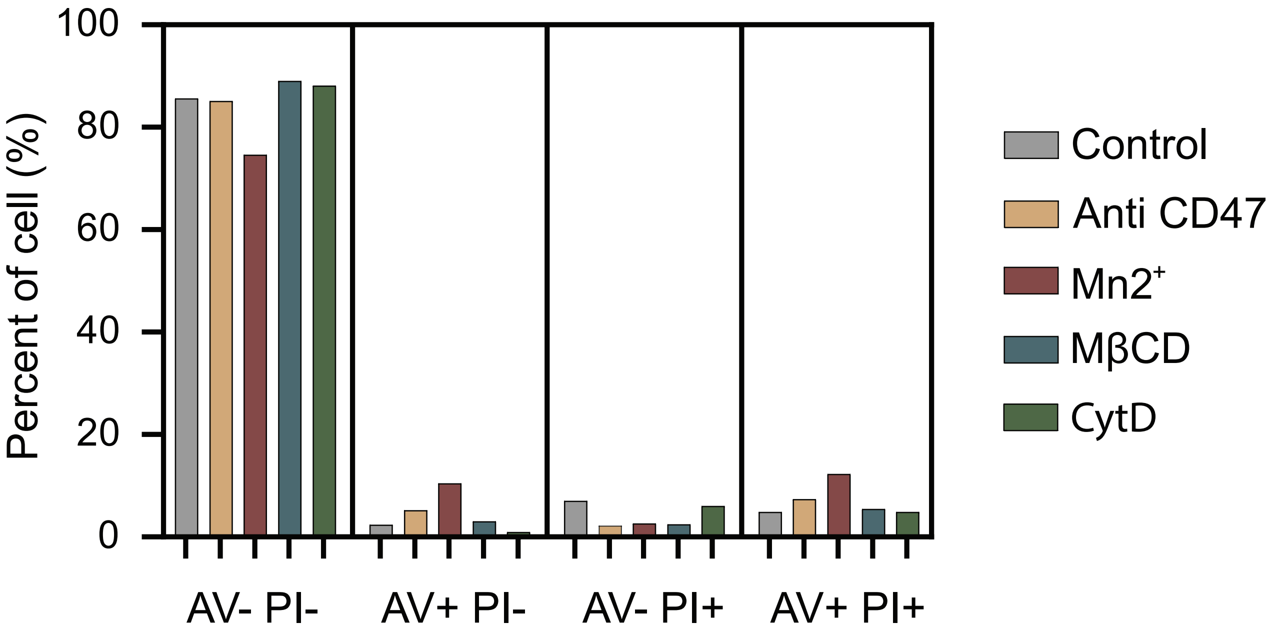

Figure S9. HeLa cells apoptosis after the treatments applied in phagocytosis assays.

HeLa cells were incubated with Cytochalasin D (CytD), Mn2+, MβCD or anti CD47 antibody (B6H12) as performed in phagocytosis experiment (Fig.5c). Cells were next incubated with Annexin V FITC (AV) and Propidium iodide (PI). For each condition, about 10000 cells were analyzed by flow cytometry. AV-PI-: viable cells; AV+ PI-: early apoptotic cells; Av- PI+: necrotic cells and AV+ PI+: late apoptotic cells.

a

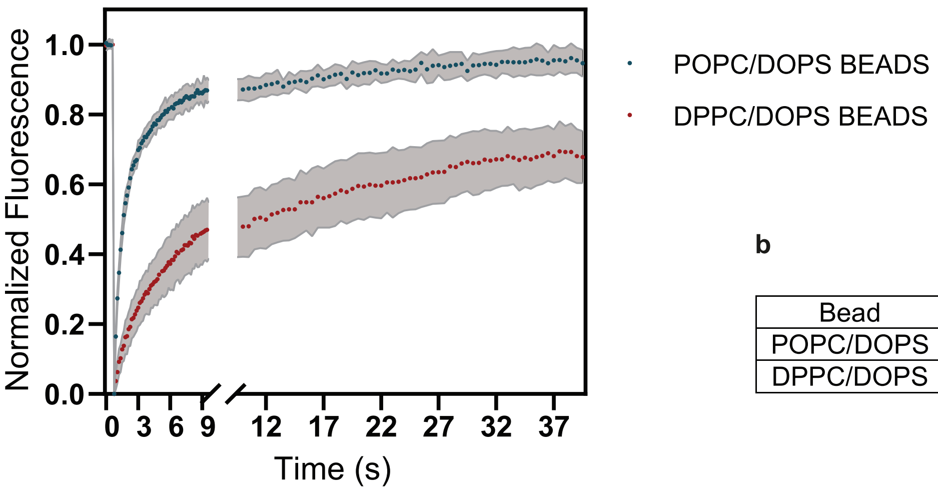

b

Bead	$T_{1/2}$ (s)	Mobile fraction	N
POPC/DOPS	0.82 ± 0.1	0,79 ± 0.08	10
DPPC/DOPS	7,05 ± 3.5	0,95 ± 0.02	11

Figure S10. Measure of TopFluorPS mobility within POPC or DPPC lipid bilayer coated silica beads by Fluorescence recovery after photobleaching (FRAP).

Silica beads were coated with lipids at the following concentration: 1% TopFluorPS, 10% DOPS, 89% POPC or DPPC. (a) The mobility of the fluorescent PS analog TopFluorPS was measured by FRAP microscopy on a spinning disk microscope.The recovery curves are presented with gray area for standard deviation (SD). (b) The T1/2 and the mobile fraction were estimated after fitting of the curves. The normalized fluorescence value 1 corresponds to the fluorescence before photobleaching; N is the number of beads.
