# Supplementary material for: Nanoscale imaging of CD47 informs how plasma membrane modifications shape apoptotic cell recognition
Source: Commun Biol. 2023 Feb 22;6:207. doi: 10.1038/s42003-023-04558-y (PMC9947010; doi:10.1038/s42003-023-04558-y)
Supplement: Supplementary file 3 — Description of Additional Supplementary Files [file 42003_2023_4558_MOESM3_ESM.pdf]

Description of Additional Supplementary Files

File Name: Supplemental Movie 1

Description: SPT αvβ3/CD47 full frame (Single Particle Tracking of integrin αvβ3 and CD47) on the surface membrane of a HeLa viable cell represented in Figure 4b. The movie contains 500 images at 12 images/s. Each image corresponds to a 34 ms exposure time. Tracks containing at least 10 localizations and reconstructed with SWIFT are shown on the left. The corresponding localizations are displayed on the right. The square delimits the zone enlarged in Movie#2. Analysis of the two-color tracking data reveals that 3.4 % of the αvβ3 tracks and 1.9% of the CD47 tracks show partial co-diffusing patterns. The overall rare observation of co-diffusion in fact can be fully explained by the low labeling density of the two partners which is required to properly track single diffusing molecules.

File Name: Supplemental Movie 2

Description: Zoomed view of single particle tracking of integrin αvβ3 and CD47 as delimited in Movie #1.
