# Supplementary material for: Nanoscale imaging of CD47 informs how plasma membrane modifications shape apoptotic cell recognition
Source: Commun Biol. 2023 Feb 22;6:207. doi: 10.1038/s42003-023-04558-y (PMC9947010; doi:10.1038/s42003-023-04558-y)
Supplement: Supplementary file 6 — Reporting Summary [file 42003_2023_4558_MOESM6_ESM.pdf]

nature portfolio

Corresponding author(s): Frachet Philippe

Last updated by author(s): Jan 23, 2023

Reporting Summary

Nature Portfolio wishes to improve the reproducibility of the work that we publish. This form provides structure for consistency and transparency in reporting. For further information on Nature Portfolio policies, see our Editorial Policies and the Editorial Policy Checklist.

Statistics

For all statistical analyses, confirm that the following items are present in the figure legend, table legend, main text, or Methods section.

n/a	Confirmed	
☐	☒	The exact sample size (*n*) for each experimental group/condition, given as a discrete number and unit of measurement
☐	☒	A statement on whether measurements were taken from distinct samples or whether the same sample was measured repeatedly
☐	☒	The statistical test(s) used AND whether they are one- or two-sided<br>*Only common tests should be described solely by name; describe more complex techniques in the Methods section.*
☒	☐	A description of all covariates tested
☒	☐	A description of any assumptions or corrections, such as tests of normality and adjustment for multiple comparisons
☐	☒	A full description of the statistical parameters including central tendency (e.g. means) or other basic estimates (e.g. regression coefficient) AND variation (e.g. standard deviation) or associated estimates of uncertainty (e.g. confidence intervals)
☐	☒	For null hypothesis testing, the test statistic (e.g. *F*, *t*, *r*) with confidence intervals, effect sizes, degrees of freedom and *P* value noted<br>*Give P values as exact values whenever suitable.*
☒	☐	For Bayesian analysis, information on the choice of priors and Markov chain Monte Carlo settings
☒	☐	For hierarchical and complex designs, identification of the appropriate level for tests and full reporting of outcomes
☒	☐	Estimates of effect sizes (e.g. Cohen's *d*, Pearson's *r*), indicating how they were calculated

Our web collection on statistics for biologists contains articles on many of the points above.

Software and code

Policy information about availability of computer code

Data collection	All data collected at M4D cell imaging platform, ISBG<br>For SR microscopy Metamorph Molecular Devices RRID:SCR_002368<br>For confocal microscopy , All samples were visualized under a laser spinning-disk confocal microscope (Olympus & Andor<br>For Flow cytometry, MACSQuant VYB cytometer
Data analysis	MACSQuantify software Miltenyi Biotec RRID:SCR_020943<br>Fiji https://fiji.sc/ RRID:SCR_002285<br>Prism GraphPad 8 RRID:SCR_002798<br>Imaris Oxford instruments RRID:SCR_007370<br>SWIFT http://bit.ly/swifttracking<br>Coloc Tesseler https://github.com/flevet/Coloc-Tesseler<br>SR-Tesseler https://github.com/flevet/SR-Tesseler<br>Metamorph Molecular Devices RRID:SCR_002368

For manuscripts utilizing custom algorithms or software that are central to the research but not yet described in published literature, software must be made available to editors and reviewers. We strongly encourage code deposition in a community repository (e.g. GitHub). See the Nature Portfolio guidelines for submitting code & software for further information.

Data

Policy information about availability of data

All manuscripts must include a data availability statement. This statement should provide the following information, where applicable:

- Accession codes, unique identifiers, or web links for publicly available datasets
- A description of any restrictions on data availability
- For clinical datasets or third party data, please ensure that the statement adheres to our policy

Complete imaging datasets are available from the Lead Contact. Data used in the figures are available on figshare DOI: 10.6084/m9.figshare.21915318

Field-specific reporting

Please select the one below that is the best fit for your research. If you are not sure, read the appropriate sections before making your selection.

☒ Life sciences ☐ Behavioural & social sciences ☐ Ecological, evolutionary & environmental sciences

For a reference copy of the document with all sections, see nature.com/documents/nr-reporting-summary-flat.pdf

Life sciences study design

All studies must disclose on these points even when the disclosure is negative.

Sample size	The number of cells analyzed in each experiment was as much as possible, depending on the technique used and this is specified in the legends figures and MM
Data exclusions	n/a
Replication	each experiment was performed at least two times independently
Randomization	*Describe how samples/organisms/participants were allocated into experimental groups. If allocation was not random, describe how covariates were controlled OR if this is not relevant to your study, explain why.*
Blinding	*Describe whether the investigators were blinded to group allocation during data collection and/or analysis. If blinding was not possible, describe why OR explain why blinding was not relevant to your study.*

Reporting for specific materials, systems and methods

We require information from authors about some types of materials, experimental systems and methods used in many studies. Here, indicate whether each material, system or method listed is relevant to your study. If you are not sure if a list item applies to your research, read the appropriate section before selecting a response.

Materials & experimental systems

n/a	Involved in the study	
☐	☒	Antibodies
☐	☒	Eukaryotic cell lines
☒	☐	Palaeontology and archaeology
☒	☐	Animals and other organisms
☒	☐	Human research participants
☒	☐	Clinical data
☒	☐	Dual use research of concern

Methods

n/a	Involved in the study	
☒	☐	ChIP-seq
☐	☒	Flow cytometry
☒	☐	MRI-based neuroimaging

Antibodies

Antibodies used

Chicken polyclonal anti calreticulin Thermofisher PA1-902A
Annexin V conjugated to Alexa Fluor 647 Biolegend 640912
Annexin V conjugated to Biotin Biolegend 640904
Rabbit polyclonal anti ERp57 Abcam ab10287
Mouse anti CD47 conjugated to Alexa Fluor A647 (Clone B6H12) Santa Cruz sc-12730 AF647
Mouse anti CD47 (Clone B6H12) Santa Cruz sc-12730
Rabbit polyclonal anti integrin beta 3 sigma AB2984
Normal mouse IgG1 Santa Cruz sc-3877
Rabbit Polyclonal anti Chicken labeled with Alexa Fluor 532 Cohesion Biosciences CSA3314
F(ab')2 Goat polyclonal anti-Mouse labeled with Alexa Fluor 488 Thermofisher A-11017

goat anti-mouse antibody labeled with CF680 (Sigma-Aldrich, SAB4600199

goat anti-mouse antibody labeled with CF680, Sigma-Aldrich, SAB4600199
Goat polyclonal anti-mouse labeled with Alexa Fluor 647 Thermofisher A21236
Donkey polyclonal anti Chicken labeled with DyLight 488 JacksonImmunoResearch 703-485-155
Goat polyclonal anti Chicken labeled with Alexa Fluor 555 Thermofisher A21437
Donkey polyclonal anti Rabbit IgG labeled with FluoProbes 647H Interchim FP-SC5110
Streptavidin conjugated to Alexa Fluor 647 Thermofisher S32357
Goat polyclonal anti-Rabbit labeled with Peroxidase Sigma A0545
Goat anti-mouse antibody labeled with CF680 Sigma-Aldrich, SAB4600199

Validation	Antibodies were used as described by the suppliers, or under conditions that are described in detail in the MM

Eukaryotic cell lines

Policy information about cell lines

Cell line source(s)	cell lines were provided by the ATCC.
Authentication	HeLa (CCL2) and J774 (TIB 67)
Mycoplasma contamination	The cells were tested forMycoplasma contamination (Mycoalert detection kit, Lonza)
Commonly misidentified lines (See ICLAC register)	*Name any commonly misidentified cell lines used in the study and provide a rationale for their use.*

Flow Cytometry

Plots

Confirm that:

☒ The axis labels state the marker and fluorochrome used (e.g. CD4-FITC).

☒ The axis scales are clearly visible. Include numbers along axes only for bottom left plot of group (a 'group' is an analysis of identical markers).

☒ All plots are contour plots with outliers or pseudocolor plots.

☒ A numerical value for number of cells or percentage (with statistics) is provided.

Methodology

Sample preparation	cell lines
Instrument	Flow cytometry analyses were performed with MACSQuant VYB cytometer
Software	MACSQuantify software (Miltenyi Biotec) . dot plot /histograms were represented with Graphpad Prism 8.
Cell population abundance	at least 10000 events in the analysis gate were collected
Gating strategy	FSC/SSC gating

☒ Tick this box to confirm that a figure exemplifying the gating strategy is provided in the Supplementary Information.
